# A Method for Biomarker Directed Survival Prediction in Advanced Non-Small-Cell Lung Cancer Patients Treated with Carboplatin-Based Therapy 

**DOI:** 10.3390/jpm3030251

**Published:** 2013-09-12

**Authors:** Wei Chen, Gerold Bepler

**Affiliations:** Karmanos Cancer Institute, Department of Oncology, Wayne State University, Detroit, MI 48201, USA; E-Mail: beplerg@karmanos.org

**Keywords:** biomarkers, cancer, protein expression, methodology, non-small-cell lung cancer, ERCC1

## Abstract

Platinum-based chemotherapy is a primary treatment of choice for advanced non-small-cell lung cancer (NSCLC). Analytical methods to specifically evaluate biomarkers predictive of therapeutic efficacy have not been developed. Two randomized phase III trials of carboplatin-based chemotherapy in advanced NSCLC were used for learning and validating the predictive value of ERCC1 *in situ* protein levels, as measured by accurate quantitative analysis (AQUA). A novel Bayesian method was applied to identify the outcome-based threshold in the learning trial only. Overall survival (OS) was assessed by Kaplan-Meier analysis with log rank testing to determine statistical significance in the validating trial. For patients treated with gemcitabine and carboplatin, the median OS was 9.5 months (95% CI 6.7 to 11.8) for the high ERCC1 group compared to 15.6 months (95% CI 11.6 to 24.8) for the low ERCC1 group in the validation trial (log rank *p*-value = 0.007). The hazard ratio for low ERCC1 was 0.598 (95% CI, 0.394 to 0.908; *p* = 0.016) relative to high ERCC1 adjusted for age, sex, and histology. Conclusions: Patients with advanced NSCLC could be stratified into high and low ERCC1 expression groups. Patients with low levels benefited from platinum-based chemotherapy, whereas those with high levels did not.

## 1. Introduction

Exploring the path towards personalized medicine is a clear need for improved selection of the most beneficial therapy for patients with non-small-cell lung cancer (NSCLC), whose five-year overall survival (OS) rate has remained at only 16% [1] for the past three decades. Although erlotinib or crizotinib are approved by the Food and Drug Administration (FDA) with companion diagnostics for advanced NSCLC patients with adenocarcinoma with certain EGFR mutations or ALK gene rearrangements, drug sensitive EGFR mutations are found in only 10% of Caucasian patients and ALK gene rearrangements in 2%–7% in the United States [2,3]. Platinum-based combination chemotherapy remains the current standard therapy for the majority of patients with advanced disease and for those undergoing adjuvant therapy and treatment concurrent with radiation. The development of predictive biomarkers able to identify NSCLC patients who are most likely to benefit from platinum-based chemotherapy would be of significant clinical importance.

The tumoral levels of expression of the excision repair cross-complementation group 1 gene (ERCC1) is a highly promising marker for response prediction to platinum agents, in particular in patients with NSCLC, as evidenced by several high-profile publications [4,5]. High ERCC1 levels are associated with resistance to platinum-based chemotherapy, while low levels are associated with sensitivity [5,6,7,8]. Despite the multiple retrospective analyses of trials on the predictive role of ERCC1 [5,9,10,11,12], there is a lack of published prospective marker based Phase III clinical data to validate ERCC1 for individualized treatment. Although a recently published reanalysis of the International Adjuvant Lung Trial (IALT) suggests that global non-isoform specific ERCC1 expression analysis may be insufficient to leverage this biomarker to predict clinical efficacy, the study clearly reconfirmed the strong link between ERCC1 (isoform 202) and platinum efficacy [13]. A recently published randomized international phase III trial of ERCC1 and RRM1 expression-based chemotherapy *versus* gemcitabine/carboplatin in advanced NSCLC [14] was the first to study the chemotherapy selection based on marker status. The trial demonstrated feasibility of the approach but showed no advantage to using the personalized compared to the standard-of-care approach. However, because the trial had a build-in internal control by design, it was demonstrated that this result is most likely a false negative conclusion.

On the basis of biological knowledge on ERCC1 and the availability of prospectively collected clinical outcomes data, we chose ERCC1 as a representative biomarker with continuous levels of expression to develop an analytical methodology with the goal to assess predictive information and to facilitate implementation of personalized therapy. A fluorescent-based immunohistochemistry (IHC) method combined with accurate quantitative analysis (AQUA) [15], which allows for automated analysis of *in situ* protein levels, had been successfully used in identifying biomarkers in tumor tissues from NSCLC patients [4,6,16,17,18,19,20,21]. One of the essential steps for developing an applicable biomarker is the determination of cut-off values for categorization of patients into marker positive (or high) and negative (or low) groups for the treatment assignment. We recently developed a robust and reproducible tool to model the relationship between the continuous biomarker and outcomes and simultaneously identifying the threshold for marker high/low groups using Adaptive Bayesian Model Selection (ABMS) [22]. Here, we applied this novel ABMS method using a learning phase III trial [6] and validated the role of ERCC1 using the data from the recent published negative phase III trial [14]. Our results suggested that ERCC1 is a predictive marker and the optimal threshold to predict treatment outcomes is lower than what was used in either prospective trial.

## 2. Methods

### 2.1. Patients and Phase III Trials

Trial NCT00190710 [6] was conducted in patients with previously untreated stage IIIB/IV NSCLC, a performance of status (PS) of 2, and measurable disease by Response Evaluation Criteria In Solid Tumors (RECIST). Patients were randomized 1:1 to a control arm of single-agent gemcitabine (G) or an experimental arm of gemcitabine and carboplatin (GC). A total of 170 patients were randomized between March, 2004, and December, 2006. The trial was terminated due to low patient accrual. Sixty-nine patients had available protein expression levels of ERCC1 or ribonucleotide reductase M1 (RRM1). There were no significant differences between the groups of patients with and without biomarker data (Table 1 in Reynolds *et al.* [6])

A randomized phase III study (NCT00499109) [14] was conducted to assess if chemotherapy selection based on tumoral protein levels of ERCC1 and RRM1 would improve survival in patients with advanced NSCLC. Chemo-naive patients with PS 0–1, measurable disease, and formalin-fixed and paraffin-embedded (FFPE) tumor specimens were eligible. ERCC1 and RRM1 were analyzed by AQUA and categorized as high or low using predetermined protein values. Patients were randomized 2:1 to the experimental arm, which received G and C for ERCC1/RRM1 low, docetaxel (D) and C for RRM1 high and ERCC1 low, G and D for RRM1 low and ERCC1 high, and D and vinorelbine (V) for RRM1/ERCC1 high, or the control arm, which received GC. The trial was designed to demonstrate a 32% improvement in six-month progression-free survival (PFS). Accrual started in May, 2007, and completed in December, 2010. A total of 275 eligible patients were randomized (Table 1 in Bepler *et al.* [14]).

### 2.2. Protein Expression Analysis

For NCT00190710, FFPE tumor blocks were shipped to a central laboratory. Full specimen sections of 4 μM thickness were cut and mounted on adhesive coated or charged glass slides. Specimens were processed in five batches of 3–20 patients each, and reference calibrator specimens for normalization of protein levels were included. Mouse clone 8F1 (1:300 dilution, lot# 9475) distributed by Sigma-Aldrich (St. Louis, MO, USA) was used for ERCC1 detection. A custom-made rabbit antiserum (R1AS-6b, 1:300 dilution, lot# 05-2007) was used for RRM1 detection [4].

For NCT00499109, FFPE tumor blocks were shipped and processed likewise by the same central laboratory. Sections were cut to 5 μM thickness and placed on frosted glass slides together with reference calibrator specimens for normalization of protein levels. Mouse clone 8F1 (lots P704, 9475, G412, H347) was used for ERCC1 detection, and rabbit antiserum R1AS-6 (lots 05-2007, 09-2008) was used for RRM1 detection.

For both trials, two full specimen sections were analyzed for each patient, one slide for RRM1 and one slide for ERCC1. For each slide, random spots (spot diameter 0.6 mm) were scanned with SpotGrabber (HistoRx, New Haven, CT, USA), and image data were analyzed with AQUA (PM-2000, version 1.2, HistoRx, New Haven, CT, USA). The maximal range of the final AQUA score is 0 to 255 (version 1.2). Since the scores were not normally distributed, we log2 transformed all scores for statistical analyses.

### 2.3. Statistical Methods

The primary objective of this study was to determine if RRM1 or ERCC1 protein levels were predictive of OS in advanced NSCLC patients treated with carboplatin-based chemotherapy. The AQUA scores of RRM1 and ERCC1 were simultaneously considered together with age (in years), sex, and histology (adenocarcinoma, squamous carcinoma, and other NSCLC) for interaction with treatment in a multi-variable Cox proportional hazards model using the ABMS method [22]. In brief, the ABMS method automatically iterates between the variable screen phase and model building phase until a decision of “best” model is made. This process greatly increases the power of identifying therapeutically targeted subgroups, while the imbedded decision rule controls the false positive rate. For each selected quantitative predictor (strong interaction with treatment) in the final model, ABMS yields posterior distribution of outcome related cut-points. The median of this posterior distribution was used as a cut-off for that quantitative predictor. This novel statistical method works well in small sample studies, due to its Bayesian properties and imbedded variable selection rules.

Data from NCT00190710 was used as a learning set. Performance status (PS) was not considered since all patients had a PS of 2. Of all patients with available protein levels for ERCC1 or RRM1, only five (7%) had stage IIIB tumors and all five were randomized to experimental arm. Therefore, the interaction between stage and treatment could not be studied. We excluded IIIB patients and focused our inference on stage IV NSCLC patients.

The log2 transformed AQUA scores from NCT00190710 were further standardized by subtracting the median and dividing by the median absolute deviation (MAD). AQUA scores from NCT00499109 were standardized in the same fashion. The cut-off identified from NCT00190710 was used to stratify patients from NCT00499109 into subgroups. Overall survival (OS) in NCT00499109 was assessed by Kaplan-Meier (KM) analysis with a log rank test to determining statistical significance. A multi-variable Cox proportional hazards model with age, sex, and histology was performed as well. A search for the optimal cut-off was performed in learning data from NCT00190710 only. Statistical tests were performed in validation data from NCT00499109 only. All reported *p*-values were two-sided. All analyses were conducted using R [23] and WinBUGS [24].

## 3. Results

### 3.1. Learning Trial NCT00190710

**Figure 1:**
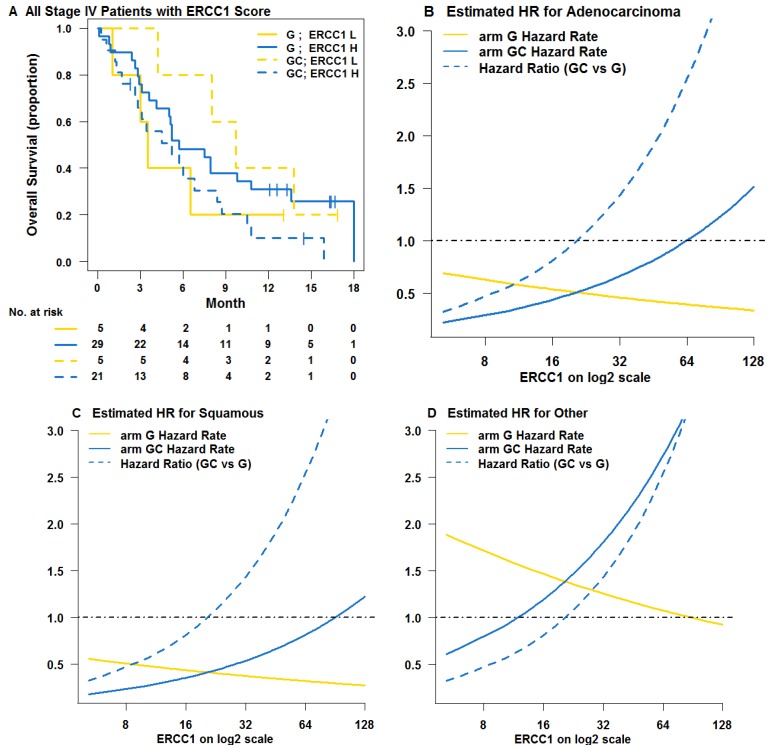
Survival and hazard ratios in the learning trial (NCT00190710) (**A**) KM curves by treatment arm and tentative high and low ERCC1 cut-offs. The median OS was 3.5 months (95% CI 3 to NA) for the low ERCC1 group and 5.7 months (95% CI 5.0 to 13.6) for the high ERCC1 group in the G arm. The median OS was 9.7 months (95% CI 8 to NA) for low ERCC1 group and 5.2 months (95% CI 2.8 to 8.7) for high ERCC1 group in the GC arm; (**B**–**D**) Estimated hazard rates and hazard ratios by histology across ERCC1 scores.

The characteristics of patients in both Phase III trials are shown in Table 1. Among the 60 stage IV patients from the learning trial NCT00190710, histology was identified as a prognostic factor. Protein levels of ERCC1 were a predictive factor, having an interaction with treatment. RRM1 was not a prognostic or predictive factor after accounting for histology and ERCC1. A cut-off led to a total of 50 out of 60 (83%) stage IV patients classified as ERCC1 high, who would not benefit from carboplatin-based treatment. The median OS of GC *vs.* G was 5.2 months (95% CI 2.8 to 8.7) *vs.* 5.7 months (95% CI 5.0 to 13.6) in the high ERCC1 group and 9.7 months (95% CI 8 to NA) *vs.* 3.5 months (95% CI 3 to NA) in the low ERCC1 group. The KM curves by subgroups are shown in Figure 1A. It is evident that when ERCC1 *in situ* protein levels increase, the estimated hazard ratio of GC *vs.* G treatment increases (Figure 1B,D). In fact, the hazard ratio changes from beneficial (<1) to harmful (>1) indicating the role of ERCC1 in predicting benefit from carboplatin.

**Table 1:** Patient characteristics in the learning (NCT00190710) and validation (NCT00499109) trials.

Variables	Learning Trial		*p*	Validation Trial		*p*
	G (n = 35) No (%)	GC (n = 34) No (%)		GC (n = 92) No (%)	GC, DC, GD, or DV (n = 183) No (%)	
Age (years)						
Median	75.6	70.8	0.414 *	63.2	64.3	0.204 *
Range	53.1–83.8	49.4–85.5		39.6–82.6	42.1–85.0	
Sex						
Male	19 (54.3)	16 (47.1)	0.633 ^†^	43 (46.7)	90 (49.2)	0.798 ^†^
Female	16 (45.7)	18 (52.9)		49 (53.3)	93 (50.8)	
Histology						
Adenocarcinoma	19 (54.3)	23 (67.6)	0.494 ^†^	47 (51.1)	99 (54.1)	0.83 ^†^
Squamous carcinoma	7 (20.0)	6 (17.6)		18 (19.6)	31 (16.9)	
Other	9 (25.7)	5 (14.7)		27 (29.3)	53 (29.0)	
Stage						
IIIB	0 (0)	5 (14.7)	0.025 ^†^	8 (8.7)	10 (5.5)	0.312 ^†^
IV	35 (100)	29 (85.3)		84 (91.3)	173 (94.5)	
ERCC1						
Median	33.4	36.7	0.72 *	68.4	79.7	0.042 *
Range	5.2–127.6	12.8–131.3		9.4–255.8	13.2–255.3	
RRM1						
Median	39.1	32.9	0.146 *	93	78.7	0.319 *
Range	5.2–90.1	6.4–105.6		4.3–248.8	2.9–255.0	

Abbreviations: G, Gemcitabine; GC, gemcitabine/carboplatin; DC, docetaxel/carboplatin; GD, gemcitabine/docetaxel; DV, docetaxel/vinorelbine; * Wilcoxon rank-sum test; ^†^ Fisher’s exact test.

### 3.2. Testing Trial NCT00499109

The threshold for ERCC1 was then used to stratify the 256 stage IV patients in NCT00499109. Thus, 202 (78.9%) patients were classified as having high ERCC1 levels in this report, which was significantly higher (McNemar test *p* value < 0.001) than the number of high ERCC1 originally defined in the testing trial 136 (53%). Among the 256 stage IV patients, 59/83 (71.1%) in the control and 143/173 (82.7%) in the experimental arm were ERCC1 high by our approach. The control arm had a significantly (Fisher’s exact test *p* value = 0.048) lower percentage of ERCC1 high patients than the experimental arm. This may partially explain the negative results from the original report of NCT00499109.

**Figure 2:**
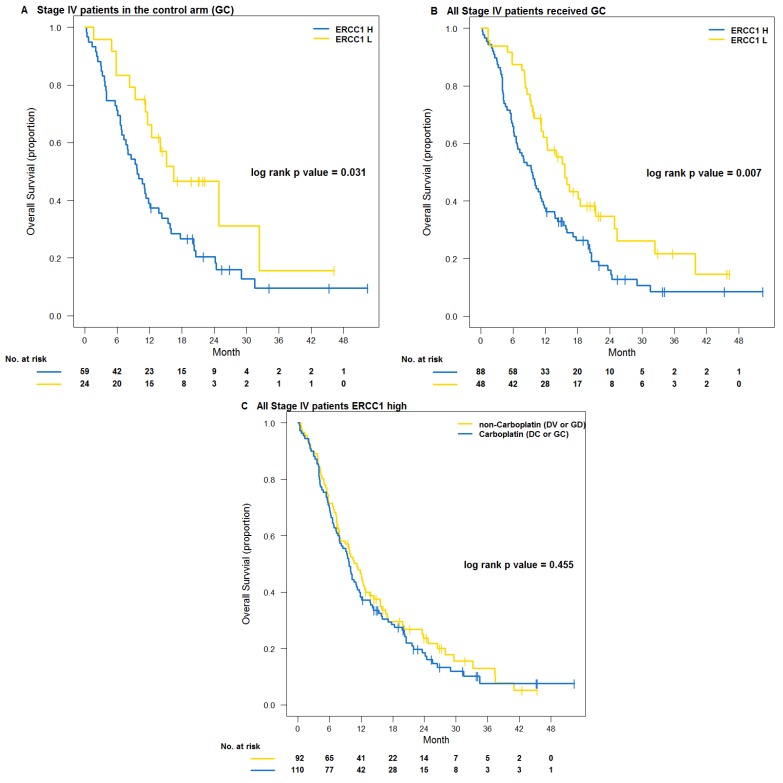
KM survival estimates in the validation trial (NCT00499109) (**A**) OS among stage IV patients in the control arm (GC treatment). The median OS was 9.7 months (95% CI 7.2 to 14.3) for the high ERCC1 group and 16.4 months (95% CI 11.6 to NA) for the low ERCC1 group (log-rank *p*-value 0.031). The adjusted hazard ratio from Cox model for the low *vs.* high ERCC1 group was 0.53 (95% CI, 0.29 to 0.97; *p* = 0.040); (**B**) OS among all stage IV patients who received GC. The median OS was 9.5 months (95% CI 6.7 to 11.8) for the high ERCC1 group and 15.6 months (95% CI 11.6 to 24.8) for the low ERCC1 group (log-rank *p*-value 0.007). The adjusted hazard ratio from Cox model for the low *vs.* high ERCC1 group was 0.598 (95% CI, 0.394 to 0.908; *p* = 0.016); (**C**) OS among stage IV patients with high ERCC1 levels. The median OS was 9.7 months (95% CI 7.9 to 11.8) for patients receiving DC or GC carboplatin-based; and 11.2 months (95% CI 7.9 to 14.4) for those receiving DV or GD non-carboplatin-based (log-rank *p*-value 0.455). The adjusted hazard ratio from Cox model for the carboplatin *vs.* non-carboplatin group was 1.04 (95% CI, .76 to 1.42; *p* = 0.799).

The characteristics of patients in the high and low ERCC1 groups are shown in Table 2. All patients in the control arm received GC regardless of marker levels. The KM curves for stage IV patients in the control group are shown in Figure 2A. The median OS was 9.7 months (95% CI 7.2 to 14.3) *vs.* 16.4 months (95% CI 11.6 to NA) for the high *vs.* low ERCC1 groups (log-rank *p*-value 0.03). The hazard ratio from a Cox model adjusted for age, sex, and histology for patients in the low ERCC1 group was 0.53 (95% CI, 0.29 to 0.97; *p* = 0.04) compared to the high ERCC1 group.

**Table 2:** Patient characteristics in the high and low ERCC1 groups of the validation trial.

Variables	All Patients		*p*	Stage IV Patients		*p*
	Low ERCC1 (n = 58) No (%)	High ERCC1 (n = 216) No (%)		Low ERCC1 (n = 54) No (%)	High ERCC1 (n = 202) No (%)	
Age (years)						
Median	64.6	63.7	0.872 *	64.6	63.8	0.924 *
Range	42.6–85.0	39.6–83.8		42.6–85.0	39.6–83.8	
Sex						
Male	28 (48.3)	105 (48.6)	1.000 ^†^	25 (46.3)	97 (48.0)	0.879 ^†^
Female	30 (51.7)	111 (51.4)		29 (53.7)	105 (52.0)	
Histology						
Adenocarcinoma	34 (58.6)	112 (51.9)	0.607 ^†^	31 (57.4)	107 (53.0)	0.800 ^†^
Squamous carcinoma	8 (13.8)	41 (19.0)		8 (14.8)	38 (18.8)	
Other	16 (27.6)	63 (29.2)		15 (27.8)	57 (28.2)	
Stage						
IIIB	4 (6.9)	14 (6.5)	1.00 ^†^	0 (0)	0 (0)	NA
IV	54 (93.1)	202 (93.5)		54 (100)	202 (100)	
ERCC1						
Median	25.1	94.2	<0.001 *	25.1	94.7	<0.001 *
Range	9.4–38.4	39.4–255.8		9.4–38.4	39.4–255.8	
RRM1						
Median	43.8	93.5	<0.001 *	44.8	92.9	<0.001 *
Range	2.9–233.9	7.7–255.0		2.9–233.9	7.7–255.0	

* Wilcoxon rank-sum test; ^†^ Fisher’s exact test.

Pooling patients who received GC from the control and experimental arms strengthened the evidence with the log-rank *p*-value changing from 0.03 to 0.007 (Figure 2B). The median OS was 9.5 months (95% CI 6.7 to 11.8) for patients in the high ERCC1 group and 15.6 months (95% CI 11.6 to 24.8) for those in the low ERCC1 group. The hazard ratio from Cox model adjusted for age, sex, and histology for patients in the low ERCC1 group was 0.598 (95% CI, 0.394 to 0.908; *p* = 0.016) compared to the high ERCC1 group.

We pooled all eligible patients who received GC or DC into a carboplatin-based treatment group and those who received GD or DV into a non-carboplatin-based treatment group. This allowed us to test the treatment effect of carboplatin *vs.* non-carboplatin based groups for patients with high ERCC1 levels. For patients with low ERCC1 levels, this comparison was not performed. All of such patients (except for three patients) received carboplatin-based regiment, because of the concept and design of the NCT00499109 trial. Figure 2C shows KM curves of OS among stage IV patients who had high ERCC1 levels. The median OS was 9.7 months (95% CI 7.9 to 11.8) for the carboplatin treatment group and 11.2 months (95% CI 7.9 to 14.4) for the non-carboplatin treatment group (log-rank *p*-value 0.455). The adjusted hazard ratio was 1.04 (95% CI, .76 to 1.42; *p* = 0.799) for the carboplatin to the non-carboplatin based group. Hence, there is no evidence that carboplatin-based regimens prolong OS for patients with high ERCC1 levels compared to non-carboplatin-based regimens.

## 4. Discussions

ERCC1 has a strong scientific rationale and has been widely studied as a prognostic and predictive biomarker. However, clinical implementation of ERCC1 into chemotherapy selection remains a relevant clinical research question with the appropriate biomarker assay still in question and the lack of an optimal cut-off point.

The current study describes the predictive role of ERCC1 as determined by the 8F1 monoclonal antibody and AQUA technology in patients with stage IV NSCLC using a novel analytical methodology specifically developed for the assessment of biomarkers with continuous values (ABMS). As this automated analysis reduces reader-related biases and generates continuous values for biomarkers, it enabled us to perform an outcomes-based threshold analysis using ABMS in a learning cohort. The identification of an outcome related cut point with the ABMS method has a clear advantage over an arbitrary cut point selection, which has traditionally been the median value. Nonetheless, these outcomes-based thresholds, or supervised learning, might increase the risk of false positive results. The number of patients from NCT00499109 was large enough to be considered as an important independent validation study of ERCC1. The outcomes-based cut-off was able to stratify patients into good or poor prognosis for OS when treated with GC to a significant degree (*p* = 0.007) in the validation trial.

Although our results appear to be contradictory to those recently described [13], it is important to note that Friboulet *et al*. did demonstrate that ERCC1 is unequivocally involved in repair of platinum-induced DNA adducts, albeit that this function is restricted to one of four isoforms (isoform 202). A potential interpretation of these results in the context of those described by us; *i.e.*, ERCC1 levels are predictive of platinum sensitivity only for patients with ERCC1 expression in the lowest decile, is that the relative quantities of the ERCC1 isoforms may differ among the spectrum of whole ERCC1 levels with higher relative isoform 202 levels in the low range of whole ERCC1. This would enrich for the biologically relevant isoform and thus increase the predictive utility of a non-isoform specific detection method, as was used by us.

There is no perfect study to validate potentially predictive biomarkers, since the regimen, the method used to measure the biomarkers, or the patient population may change over time. We only validated the cut-off in patients treated with GC. It is desirable to validate the cut-off for patients treated with G established in the learning trial as well, but clinical and concurrent biomarker data obtained for this regimen are not available. We considered using patients who received GD in the validation trial as a surrogate for group G. However, all 37 patients assigned to this treatment had high ERCC1 levels according to our cut-off. Patients who received DV were considered as an alternate surrogate group. Nevertheless, among the 64 patients assigned to DV, only three had low ERCC1 levels according to our cut-off. This was due to the original design of the validation trial where non-carboplatin-based regiments (GD and DV) were only used in patients with high pre-specified ERCC1 levels.

Another limitation of this study is that there were differences in the distribution of the marker levels between the two trials (Table 1). The validation trial had much higher protein levels overall. This may be a result for a variety of reasons; *i.e*., (1) the patient population (differences in eligibility criteria); (2) tissue sample collection (a standard operating procedure for tissue acquisition and fixation was not established); (3) processing of specimens (in the learning trial, specimens were processed in batches over a two-week time period while specimens in the validation trial were processed individually over a 3.5-year time period); (4) the reagents used for ERCC1 determination (different lots and vendors for the 8F1 ERCC1 antibody were used); (5) the equipment used for ERCC1 determination (although the same equipment and software was used for both trials, intensity of the light source and exposure times may impact AQUA scores when using version 1.2). It is important to note that in both trials, ERCC1 protein levels were adjusted using calibration specimens that were incorporated into each assay performed to minimize the technical variations. In this report, the optimal cut-off point of −0.879 on the standardized AQUA scores was used, which reflected the scaled distance from the median ERCC1 score in each trial. The different distributions of AQUA scores in the two trials make it difficult to introduce ERCC1 as a biomarker in a future prospective trial setting. This confirms that biomarker driven trials rely greatly on the robustness and reproducibility of marker measurements. After careful optimization of the methodology and standardization of lab technical procedures, biomarkers should be reproducible and allow for individualization of patients’ therapy.

## 5. Conclusions

In conclusion, application of our novel ABMS method enabled the identification of an out-come related cut-off point for classification of advanced NSCLC patients into carboplatin-resistant and sensitive groups. The described methodology can easily be adapted to other biomarkers and diseases, even if the number of patients in the learning set is limited. This paper strongly reinforced ERCC1 as a predictive marker of chemotherapy efficacy achieved by the new Bayesian method, taking the theoretical role of ERCC1 closer to clinical implementation.
